# Sutureless scleral fixation Carlevale IOL: a review on the novel designed lens

**DOI:** 10.1007/s10792-022-02579-w

**Published:** 2022-11-25

**Authors:** Matteo Mario Carlà, Francesco Boselli, Federico Giannuzzi, Tomaso Caporossi, Gloria Gambini, Luigi Mosca, Alfonso Savastano, Stanislao Rizzo

**Affiliations:** 1 Ophthalmology Unit, Fondazione Policlinico Universitario A. Gemelli, IRCCS, Rome, Italy; 2 Catholic University “Sacro Cuore”, Largo A. Gemelli, 8, Rome, Italy

**Keywords:** Carlevale lens, Secondary lens implant, Secondary posterior chamber lens implant, Single-piece IOL, Sutureless scleral fixation, Pseudoexfoliation (PXF), Aphakic

## Abstract

*Background*: Complicated cataract surgery is the main cause of secondary lens implantation surgery. Several approaches have been introduced to face those circumstances. As it concerns scleral-fixated IOLs for the posterior chamber, many types of IOL can be implanted. The aim of article is to review the single piece sutureless scleral fixation Carlevale lens; *Methods*: Narrative review; *Results*: Several works described as safe the IOL implantation utilizing the handshake approach, without tactile manipulation, which allows for self-centration and lens firm fixation in uncomplicated surgery. This allows to reduce high order aberration such as astigmatism and coma, with a very good postoperative BCVA *Conclusions*: Carlevale lens is one of the best option to manage insufficient capsular support.

## Introduction

Modern cataract surgery produces outstanding outcomes and a speedy visual recovery when the intraocular lens (IOL) in the capsular bag. Nevertheless, some conditions such as pseudoexfoliation (PXF), lens displacement in the vitreous cavity, post-traumatic cataract surgery, and Marfan disease may all determine an insufficient capsular support, making in-the-bag or ciliary sulcus IOL implantation impossible [1]. In this eventuality, the implantation of a IOL remains a surgical challenge, particularly given patient expectations in contemporary ophthalmic surgery. Several approaches have been introduced in order to face those circumstances such as anterior chamber IOLs (ACIOLs) or iris-fixated IOLs (IFIOLs) in the anterior chamber, or scleral-fixated IOLs for the posterior chamber (SFIOLs) [2, 3].

ACIOLs implantation in the iridocorneal angle is technically feasible, but it necessitates a large corneal or scleral incision and is frequently associated with complications such as significant induced astigmatism (IA), bullous keratopathy, transient corneal edema, uveitis and increased intraocular pressure (IOP) [4]. On the other side, iris claw lens implantation required a 5.5 mm incision to insert the IOL, which was secured without sutures posterior to the iris, thanks to tiny haptics, which granted increased stability and lesser risk of iris damage [5]. This approach offered excellent visual results, even though the wide corneal incision causes substantial postoperative astigmatism and the integrity of the iris diaphragm an essential pre-requisite of this technique [6].

The best tolerability and safety profile was provided by PC IOLs, which, thanks to the lens positioning distant from the anterior segment structures, offered the benefit of reducing the risk of post-operative side effects such as glaucoma and bullous keratopathy [7–12]. In 1997, Maggi and Maggi were the first to describe pars plana fixation of a PC IOL achieved by the transscleral passage of the haptics. Ten years after this ancillary technique, Gabor and Pavlidis introduced a sutureless procedure with a foldable hydrophilic acrylic 3-piece IOLs, put via a standard sub-2.8 mm clear corneal incision [13]. This technique required a 360° conjunctival peritomy, two 180° sclerotomies, and scleral tunnels parallel to the limbus made with a 24-G needle, where len’s haptics were placed without sutures [14].

Prasad pioneered a less invasive procedure that avoided conjunctiva peritomy, employing two extra trocars at 2 mm from sclero-corneal limbus to externalize and leave lens haptics beneath the conjunctiva, without sutures [15]. Another approach, reported by Agarwal, consisted in biological glue binding of scleral flaps [16]. During the years that followed, several variations of these surgeries were described, both with and without conjunctival opening, to simplify haptics grabbing and insertion in the scleral tunnels, as well as increasing the IOL's fixation and stability [17–19]. One example of those innovative surgical procedures was Yamane et al. flanged intrascleral double-needle fixation technique [20].

Nevertheless, one of the major drawbacks encountered in those approaches derived from the design of the commonly used 3-piece IOLs, which, although having a large optic diameter and strong and thin haptics arrangement, were not intended for intrascleral use, arising questions regarding the IOL's long-term stability and centration. The idea of a specifically designed sutureless scleral fixation IOL with hook-shaped haptics was initially suggested by Yoshida et al.[21]

A novel intraocular acrylic lens, the single-piece sutureless scleral fixation (SSF) Carlevale lens (FIL SSF, Soleko IOL Division, Italy), has recently made its market debut. This is a one-piece foldable IOL with a new design characterized by flexible sclero-corneal plugs at the ends of two haptics that are implanted and fastened to the sclera. The advocated advantages of its structure are related to the particular shape of the IOL, which is built to be suspended into the posterior chamber through the sclero-corneal plugs. Different surgical approaches have been claimed, with Veronese et al. reporting sutureless scleral fixation of Carlevale lens with plugs left beneath the conjunctiva [22], while Barca et al. previously carried out encouraging outcomes with sutureless intra-scleral fixation [23].

The introduction of this new design appeared as an important step in the context of insufficient capsular support. We fathomed the recent published researches regarding this technique, with an analysis of the following keywords: “Carlevale”, “sutureless scleral fixation”, “SSF IOL”, in order to collect a comprehensive evaluation of its effectiveness and safety profile. The goal of this review was indeed to highlight the surgical and refractive results of employing the Carlevale IOL in an SSF procedure and to compare its results with previous approaches described in literature, with the purpose to understand whether this IOL may become a landmark in PC IOLs setting.

### Lens design

The Carlevale lens is a single-piece, foldable, hydrophilic acrylic IOL with T-shaped harpoons (width 2 mm, length 1 mm) projecting from the closed haptics, to enable self-anchoring to the sclera without sutures. To provide a more natural effective lens placement, minimize iris chafing and decrease pupillary bloc risk, the haptics feature a 5° anterior angulation with regard to the optic plate. Moreover, two tiny asymmetric incisions on the haptics enable surgeons to rapidly verify correct lens unfolding since they would be in a specular location if the lens was upside down. The lens can be injected via a 2.2 or 2.7-mm corneal incision using a specialized disposable plunger injector and cartridge (MedicelViscojet 2.2 or 2.7).

**Fig. 1:**
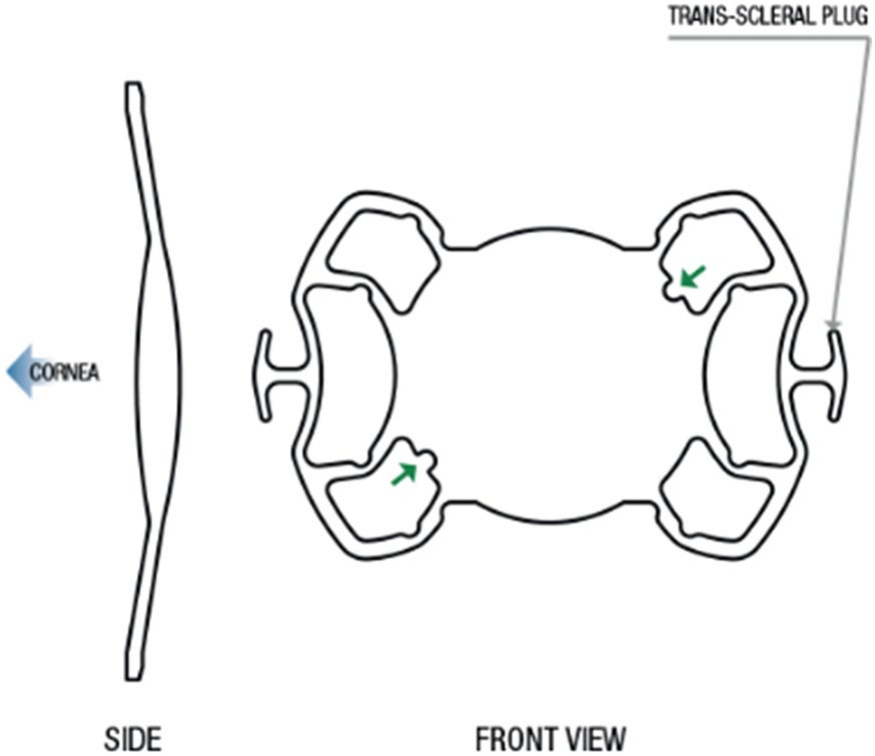
Shape and profile of one-piece foldable acrylic Carlevale lens. Image obtained from manufacturer website

Carlevale IOL has a total diameter of 13.2 mm and a 6.5 mm optical diameter, with a 25% H20 and UV filter. The refractive index of the IOL is 1.461, with diopter range varying from -5.00 to + 35.00 D, and a toric variant also available (cylinder power between 5 and 10 D, increments of 1 D). The A constant is 118.5 (Fig. 1).

### Surgical technique

After determining the corneal white to white diameter (WTW), an infusion line is inserted 3.5 mm posterior to the limbus in the inferotemporal quadrant. A minor conjunctival peritomy is performed nasally and temporally, and two straight incisions traveling posteriorly to the limbus for 2.5 mm at the 0° and 180° axes are made with a crescent blade. Within each incision, the sclera is dissected to generate two opposing pockets on each side of the incisions, and a sclerotomy is done at 1.75 mm from the limbus using a 25 or 23-gauge needle. At the 12 o'clock position, a clean corneal incision of 2.75 mm (2.2 mm for aphakia and subluxated lens) is made to implant the IOL into the eye using the injector. The Carlevale IOL is injected into the anterior chamber via the corneal tunnel using a Viscojet injector (Medical Viscojet 2.2 mm), and the leading plug is grasped with a 23-gauge crocodile tip forceps inserted into the vitreous chamber via the sclerotomy, and then externalized under the scleral flap in a single maneuver. Finally, a second forceps is introduced through the side port in order to catch the trailing plug, which is successively transferred to the first forceps and externalized using the handshake method, with no extra-intraoperative procedures required for IOL centration. Suturing of scleral incisions is accomplished using a simple butterfly or cross-stitch, with two passes perpendicular to the incision and parallel to the boundaries of the plugs, allocated within the two pockets. The two passes, placed anteriorly and posteriorly to the plug margins, close the wound and theoretically immobilize the plug. Finally, the knot is threaded through the scleral incision and secured with surgical tape. The scleral flaps and conjunctival wound can be sealed with nylon 10/0 and polyglactin 8/0 (Vicryl), respectively, or with Vycril 8/0 for both [20, 23–25].

### Surgical implantation variants

A different approach for SSL-FIL implantation was described by Fiore et al., who performed a nasal and temporal conjunctival peritomy before performing two 4-mm-thick partial thickness scleral flaps and two opposing 1-mm-deep sclerotomies with a 25-gauge needle inside the scleral bed at 1.75 mm from the limbus. A 2.2 mm clean corneal tunnel and a side port were then established, and the IOL was slowly injected into the AC while a 25-gauge crocodile-tip forceps was introduced through the sclerotomy to grip and externalize the haptic using the handshake method was performed. Finally, the scleral flap was simply overlapped to the underlying sclera without the use of sutures, and the conjunctiva was sutured above the scleral flap at the conclusion of the procedure [26].

On the other hand, Caporossi et al. recently published another surgical variation in which the two vitrectomy ports are used as lens plug fixation sites. Following the creation of the 0–180° scleral pockets, the 25 (for subluxated IOL or aphakia) or 23-gauge (for dropped lens) trocars were implanted at 1.8 to 2.0 mm from the limbus to coincide with the previously produced pockets. The Carlevale IOL was injected gently into the anterior chamber through a 2.5 mm corneal tunnel, with the leading plug grabbed with end-grasping tip forceps and delivered into the vitreous chamber via the 25–23-gauge cannula. While the forceps were still inside the vitreous chamber, the cannula was removed over the arm of the forceps, and the leading plug was externalized inside the scleral pocket in a single action. After being extracted from the cannula using the process already described, the trailing plug was grabbed with two forceps and externalized. Extra-intraoperative procedures were not required to achieve IOL centration [27].

### Clinical results

#### Refractive results

Among all investigations, Carlevale IOL implantation was performed in the following situations: dislocated PCIOL due to PXF (the most frequent), followed by dropped or subluxated IOL, crystalline lens dislocation and aphakia. A report of refractive results among all studies is shown in Table 1.

**Table 1:** Comparison between preoperative BCVA (LogMar) and post-operative BCVA (LogMar). BCVA: best corrected visual acuity; SD: standard deviation

Study	N° of eyes	Preoperative BCVA (Mean ± SD)	Postoperative BCVA (Mean ± SD)
Veronese et al	4	0.50 ± 0.33	0.08 ± 0.08
Barca et al	32	0.46 ± 0.29	0.13 ± 0.12
Georgalas et al	169	0.58 ± 0.49	0.09 ± 0.01
Caporossi et al	60	0.46 ± 0.60	0.36 ± 0.51
Rohuette et al	72	0.48 ± 0.50	0.16 ± 0.65
Rossi et al	78	0.86 ± 0.56	0.38 ± 0.42
Fiore et al	18	–	0.42 ± 0.33
Vaiano et al	54	0.93 ± 0.61	0.38 ± 0.38
Boccuzzi et al	5	0.49 ± 0.20	0.19 ± 0.10
Seknazi et al	20	–	0.23 ± 0.51

The early findings of Veronese et al. were based on 4 patients with mean preoperative best corrected visual acuity (BCVA) of 0.50 ± 0.33 logMAR (range: 1–0.3 logMAR). BCVA improved to 0.08 ± 0.08 logMAR (range: 0.2–0 logMAR) following surgery. Furthermore, there was no statistically significant difference in mean intraocular pressure (IOP) between preoperative and postoperative measurements (preoperative IOP: 16.5 ± 2.7 mmHg; postoperative IOP: 17.3 ± 3.6 mmHg) [22].

According to Barca et al., compared to preoperative values of 0.46 ± 0.29 log-MAR, mean BCVA rose to 0.22 and 0.18 log-MAR at four and eight months, respectively, and to 0.13 ± 0.12 log-MAR at twelve months, with a mean refractive prediction error of 0.24 ± 0.81 diopters (D) [23]. The mean corneal endothelial cell density decreased from 2343 cells/mm2 to 2215 cells/mm2 and 2208 cells/mm2 at 4 months and 8 months, respectively, indicating a mid-term decrease in endothelial cell density [23].

In a retrospective examination of 169 individuals, Georgalas et al. found an improvement from 0.58 ± 0.49 LogMAR to 0.09 ± 0.01 LogMAR at last follow-up (*p* = 0.0001) [28].

Similarly, Caporossi et al. analyzed 60 eyes undergoing SSF-IOL implantation. Mean BCVA at the time of surgery was 0.46 ± 0.60 logMAR, while the mean postoperative BCVA improved to 0.36 ± 0.51 logMAR after four months. According to the Sanders–Retzlaff–Kraff trial formula, the mean refractive prediction error was -0.27 ± 0.78 diopters on average [27].

Furthermore, Rouhette et al. showed that functional BCVA improved in 83.3% of the cases after implantation, with no cases of visual impairment. The mean preoperative BCVA was 3.2/10 ± 0.31, reaching 7.2/10 ± 2.1 at 6 months follow up. A few days after surgery, the spherical equivalent was 0.3 D, and this value remained constant throughout time. At the same time, there was no change in the corneal astigmatism (1.4 D preoperative vs. 1.5 D postoperative) [29].

Rossi et al. recently conducted a surgical series that involved 78 patients, focusing on eyesight improvement. They found a change from an average of 0.86 ± 0.56 logMAR to 0.38 ± 0.42 logMAR at the end of the study period, with significant visual gains at 1, 3, and 6 months follow up (*p* < 0.0001) [30].

Fiore et al. evaluated 18 eyes who underwent IOL implantation with a Carlevale lens. BCVA after surgery was 0.42 ± 0.33 logMAR, with a prediction error for refractive spherical equivalent of 0.31 ± 0.71D. In another study, Fiore analyzed the differences between two different surgical techniques for Carlevale lens (23-gauge vs. 25-gauge sclerotomies), resulting in no significant difference between the two groups for what concerns BCVA and prediction error [24].

Vaiano et al. analyzed 54 eyes with a median preoperative BCVA of 0.93 ± 0.61 logMAR undergoing Carlevale lens implantation. At three months, mean BCVA was 0.42 ± 0.34 logMAR, increasing to 0.38 ± 0.38 logMAR one year after surgery [31].

Research conducted by Boccuzzi et al. compared different IOL implantation techniques: iris-claw lenses implanted in the anterior chamber in group 1, sutureless intrascleral three-piece IOL (MA60MA, Alcon Inc.) in group 2 and transscleral IOL fixation with an intrascleral plug utilizing Carlevale's IOL (Carlevale IOL, Soleko, Italy) in group 3. No statistically significant differences regarding BCVA were found among the three groups, with a reported pre-operative and post-operative BCVA of 0.49 ± 0.2 and 0.19 ± 0.1 LogMAR in the Carlevale IOL group [25].

Seknazi et al. compared Artisan iris-claw lens (Artisan Aphakia IOL model 205, Ophtec BV, Groningen, The Netherlands) and Carlevale lens, in a retrospective research. The mean post-operative BVCA in group 1 was 0.35 ± 0.29 logMAR and 0.23 ± 0.51 LogMAR in group 2, showing no significant differences (*p* = 0.19). The mean refractive error following surgery was significantly different between the two groups: 0.99 ± 0.57 D in group 1 and 0.46 ± 0.36 D in group 2, respectively (*p* 0.01). Furthermore, mean induced astigmatism was 1.72 ± 0.96 D in group 1, and 0.72 ± 0.52 D in group 2 (*p* = 0.01) [32].

In conclusion, D’agostino et al. compared 15 eyes undergoing a three-piece IOL (ALCON MA60AC) implant and 16 eyes receiving the FIL-SSF Carlevale IOL. Unlike other studies, BCVA did not improve substantially at 6-month follow-up when compared to baseline values, and there was no difference between the two groups (*p* = 0.48). Both groups had a non-significant 1 D mean refractive error, with no difference between the two groups in the induced mean postoperative astigmatism at 3 months. When astigmatism developed after surgery, it was higher in group 1 (1.91 ± 2.07 D) than in group 2 (0.67 ± 0.88 D) and this difference was statistically significant (*p* = 0.04) [33].

#### Tilting

Thanks to its design, the Carlevale FIL-SSF IOL should be fully centered and stable, in an "anatomical" position with no contact with the iris as reported in few studies [28, 29]. Based on this assumption, some researches focused on IOL positioning and tilting. Fiore reported an IOL’s tilting of 2.2° ± 1.6° on average [24], very similar to the results reported in Barca’s study (2.08 ± 1.19° of mean tilt) [23].

In the end, Vaiano et al. reported a ì IOL tilt value of 3.1° ± 1.1° (range: 1° to 5.5°) at 12 months after surgery. Moreover, in his study IOL tilting at 12 months significantly correlated with BCVA [31].

#### Adverse events

Although the surgical technique for Carlevale lens’ implantation appears transversally affordable for both anterior and posterior segment surgeons, attention must be put on possible postoperative complications that might occur. Problems such as hypotony, malpositioning, subluxation, corneal edema associated with increased IOP, cystoid macular edema, vitreous hemorrhage and retinal tears are all possible outcomes.

No post-surgical problems, such as iatrogenic IOL distortion, IOL haptic breaking, IOL decentration, endophthalmitis, or retinal detachment, were reported in Veronesi’s study. In none of the eyes, macular optical coherence tomography (OCT) revealed cystoid macular edema (CME). The IOL haptics remained well seated inside the sclera, with no conjunctival erosion or local inflammatory reaction [22].

In Barca’s case series, two months after surgery one eye (3.1%) developed temporary CME, which was effectively treated with nonsteroidal anti-inflammatory eye drops. Pigment dispersion with AS-OCT findings of reverse pupillary block was observed 1 week after surgery in 2 eyes (6.2%), and in both cases, YAG peripheral iridotomy was sufficient to restore the physiological iris profile. After 7 months, one patient (3.1%) developed intraocular hypertension due to pigment dispersion, necessitating the use of anti-glaucoma eye drops. One eye (3.1%) had a self-limited vitreous hemorrhage. There was no postoperative hypotony in any of the eyes, plug externalization or conjunctival erosion or retinal degeneration during an 8-month minimum follow-up period [23].

Diversely, Georgalas et al. reported a transitory increase in IOP in 16.5% of cases, even if the mean IOP did not change significantly (14.9 mmHg vs. 14.8 mmHg). All these patients had pseudo-exfoliation and were on anti-glaucoma medication before surgery, and their IOP was well-controlled. There was no evidence of retinal detachment, any other retinal/macular pathology or corneal or haptips’ problems in any case. Mild vitreous bleeding was found in eight eyes (4.7%) in the initial post-operative period, which spontaneously cleared within three weeks [28].

Caporossi et al. reported four eyes with early postoperative hypotony (6.6%) and only two cases of CME (2.8%) were discovered after three months, both regressing within the sixth month thanks to nonsteroidal anti-inflammatory eye drops. Differently from Georgalas, post-operative IOP raise was not reported in any patient. [27] Rouhette, on the other side, found out that 30% of patients had IOP less than 10 mmHg in the first week with a single case of retinal detachment that occurred at 3 months and was successfully treated [29].

Rossi reported 4 cases (5.1%) of CME, 2 retinal tears (2.5%), 2 retinal detachments requiring surgery (2.5%), 2 oculoplastic complications (2.5%), 2 cases of ocular hypertension requiring drops (2.5%), and 1 case (1.2%) of corneal decompensation requiring DSAEK [30].

The most frequent intraoperative complication reported by Vaiano et al. was the rupture of the IOL haptics, which happened in 6 eyes (11%), in most cases during the extraction through the scleral surface with damage of the distal T-shaped plugs. In the other cases, the problem was the malposition in the cartridge, with a proximal damage. Early problems included transient corneal edema in 5 patients (9.25%), a minor anterior chamber inflammatory reaction in 4 eyes (7.4%), and mild intraocular bleeding in 4 eyes (7.4%). There were no incidences of ocular hypotony; however, there were two instances of ocular hypertension (3.7%). Late complications included 2 occurrences of exposed haptics beneath the conjunctiva (3.7%) and 4 cases of macular edema (7.4%). Three months following the treatment, there was one retinal detachment (1.85%), this time in a patient with Marfan syndrome. One retinal detachment and one epiretinal membrane development accounted for the two occurrences of decreased visual acuity (3.70%). There were no incidents of IOL dislocation or postoperative endophthalmitis [31]. In Fiore’s research, instead, no complications such as iatrogenic IOL dislocation, IOL haptic rupture, IOL decentration, intraocular pressure rise, or endophthalmitis, were reported [24, 26].

Boccuzzi described a case of IOL malposition and subluxation in both the transscleral flanged and iris-claw groups, with the second needing further surgery. Two eyes had corneal edema as a result of elevated IOP that persisted more than seven days, solved thanks to topical antiglaucoma therapy. In the iris-claw group, three eyes had pupillary abnormalities due to incorrect iris hooking, even if none of them necessitated IOL relocation. Vitreous hemorrhage occurred in two eyes (one each in the transscleral and Carlevale's groups), probably as a result of near-limbus sclerotomy. Seven eyes, all from the iris-claw group, suffered from severe astigmatism (> 3D) after surgery. This was addressed by removing the major incision sutures sequentially over time, even if one patient's astigmatism remained > 3D until the final follow-up [25].

In iris-claw lenses implants, two eyes suffered IOL dislocation, three eyes developed cystoid macular edema and one eye had a large vitreous hemorrhage that necessitated a second surgical treatment. In Carlevales’ group two eyes (10%) experienced cystoid macular edema, one eye had a mild vitreous hemorrhage that resolved spontaneously after one month of follow-up, one eye had a neurotrophic ulcer that resolved after treatment with lubricating eye drops, and one eye had a broken plug during surgery, necessitating immediate IOL removal and the implantation of a new lens of the same model [32].

A review of adverse effects among all studies is available in Table 2.

**Table 2:** Comparison between all the major postoperative complications. RD = retinal detachment

	Elevated IOP	Hypotony	Malposition	Subluxation	Corneal decompensation	Cystoid macular edema	Hemorrhage	Retinal tears/RD	Endophtalmitis
Veronese et al	0 (0%)	0 (0%)	0 (0%)	0 (0%)	0 (0%)	0 (0%)	0 (0%)	0 (0%)	0 (0%)
Barca et al	2 (6,2%)	0 (0%)	0 (0%)	0 (0%)	0 (0%)	1 (3,1%)	1 (3,1%)	0 (0%)	0 (0%)
Georgalas et al	28 (16.6%)	0 (0%)	0 (0%)	0 (0%)	0 (0%)	0 (0%)	8 (4,7%)	0 (0%)	0 (0%)
Caporossi et al	0 (0%)	4 (6,6%)	0 (0%)	0 (0%)	0 (0%)	2 (2,8%)	0 (0%)	0 (0%)	0 (0%)
Rohuette et al	0 (0%)	21 (29%)	0 (0%)	0 (0%)	0 (0%)	0 (0%)	0 (0%)	1 (1,3%)	0 (0%)
Rossi et al	2 (2,5%)	0 (0%)	0 (0%)	0 (0%)	1 (1,2%)	0 (0%)	4 (5,1%)	2 (2,5%)	0 (0%)
Fiore et al	0 (0%)	0 (0%)	0 (0%)	0 (0%)	0 (0%)	0 (0%)	0 (0%)	0 (0%)	0 (0%)
Vaiano et al	2 (3,7)	0 (0%)	0 (0%)	0 (0%)	0 (0%)	0 (0%)	4 (7,4%)	1 (3,7%)	0 (0%)
Boccuzzi et al	2 (40%)	0 (0%)	1 (20%)	0 (0%)	0 (0%)	0 (0%)	1 (20%)	0 (0%)	0 (0%)
Seknazi et al	0 (0%)	0 (0%)	0 (0%)	0 (0%)	0 (0%)	2 (10%)	0 (0%)	0 (0%)	0 (0%)

## Discussion

After 5 years of follow-up after cataract surgery, the rate of intraocular lens displacement increased from 0.1 to 3%. PEX, ocular traumatisms, vitreoretinal surgery, myopia, and uveitis are all risk factors [34, 35]. The increased frequency of secondary IOL implantation surgeries may be attributed to the growing number of pseudophakic and aphakic people [36, 37]. In situations of difficult cataract surgeries, IOLs must be implanted in new sites, such as the anterior chamber, iris, sulcus, or sclera [38].

In 2003 a comprehensive evaluation of IOL implantation without capsular support was conducted with eight secondary IOLs’ implantation techniques, such as ACIOLs, IFIOLs, and SFIOLs, but not enough data to establish the best IOL and techniques were gathered. Several studies have reported advantages and disadvantages of various lenses [39–42].

As it concerns scleral fixation’s approach, the first presented technique exploited scleral sutures. The presence of these sutures, both in 10–0 polypropylene or gore-tex like material, can cause conjunctival erosion with the associated risks of endophthalmitis, lens tilt and lens dislocation. For this reason, more attention has been put on sutureless procedures, due to the possibility of avoiding these dangers. A revolutionary sutureless IOL scleral fixating technique has been proposed by Yamane in 2017, based on externalization and cauterization of haptics of a three-piece IOL (X-70 [Santen, Osaka, Japan]; Tecnis ZA9003 [Abbott Medical Optics, Santa Ana, CA]; PN6A [Kowa, Tokyo, Japan]; or MA60MA [Alcon Laboratories, Inc]) to form tiny flanges, successfully fixing the IOL haptics in the eye wall [20, 43]. Furthermore, Agarwal et al. described a method in which the externalized IOL haptics (Aurolab, India) were fixed in partial thickness limbal scleral flaps with fibrin [44]. Another possibility was presented by Scharioth, where the haptics were put in 2 to 3 mm scleral tunnels adjacent to the sclerotomies. Furthermore, the flattened flanged intrascleral fixation technique, which is a modification of Yamane’s technique, has been reported to provide IOL stabilization, less tilt and decentration even in pediatric cases, in Marfan syndrome and in cases with zonular dialysis [45, 46].

Nowadays, the sutureless scleral fixation (SSF) technique using the Carlevale IOL it’s a revolutionary technique that preserves the conjunctiva, reduces suture-related complications, surgery time, and complexity. Carlevale IOL is a single piece of optical equipment. By using this implant, the risk of damage to the haptics is significantly reduced, since each of them is equipped with a small plug, and only the latter is grasped and pulled through the sclerotomy with the forceps, thereby reducing manipulation and maintaining the integrity of the haptics. Furthermore, thanks to the self-blocking mechanism and the harpoon-like plugs, which are protected and halted on the scleral bed underneath the sutured scleral flaps, these lenses have exceptional stability. This allows for IOL torsion and decentration rates reduction, with “natural” centration if the scleral flaps are separated by 180 degrees [22], in order to minimize optical aberrations. In contrast, other systems rely heavily on the symmetry of fixation of the haptics in the tunnel to get a satisfactory result, so Carlevale IOL is less time consuming and needs less technical skill than other sutureless procedures [16]. Only the need of scleral flap or conjunctiva closure with suture could extend the surgical time.

As it concerns the efficacy, Barca et al. report a similar prediction error between Carlevale’s IOL and other sutured IOL and SSF, such as other authors [23, 27]. Indeed, a mean tilt of 2.08 ± 1.19 degrees was reported, instead of a range between 2.53 ± 1.43 and 5.62 ± 3.86 degrees in Yamane's report and 2.9 ± 2.6 to 3.2 ± 2.7 degrees in Agarwal’s glue's IOL technique. The possible explanation relies on the limited deformation of the Carlevale IOL thanks to the reduced manipulation during surgery, bringing a more standardized position [23]. Furthermore, although the comparison among iris-claw lens, flanged transscleral fixated IOLs with Yamane technique and sutureless transscleral hook IOL fixation (Carlevale IOL), did not show variations in functional recovery in Boccuzzi’s report, Carlevale’s IOL group had better postoperative corrected vision, even if not statistically significant [25].

The stability of this type of IOL is also highlighted by the low rate of dislocation or pseudophacodonesis claimed in several studies [23]. One of the main complications of this surgery is the early hypotony due to leakage from corneal tunnel and/or sclerotomies. This complication is reported with different percentages between the several studies, from none to 30% [23, 29]. Totan, Hu, and Walsh reported that the use of 25–27-gauge trocars to guide the IOL haptic through the scleral tunnel decreases postoperative ocular hypotension [47–49]. Problems such as retinal detachment or cystoid macular edema are usually rare and reduced by the concomitant vitrectomies done in these patients [28]. The Carlevale lens lacks a spherical optic plate with projecting "J" or "C" shaped haptics, and the closed haptics are broader than the optic plate, "preserving" its margin. This appears to prevent the iris from migrating posterior to the IOL when the IOL is angled posteriorly [30]. Vitreous hemorrhage is usually limited and transient in most of the reports.

One possible application of this technique is eyes with high corneal astigmatism, due to low degree of IOL tilt and the ability to achieve good centration that bring low risk of astigmatism and coma. This is underlined by Seknazi, who found a significant difference between mean induced astigmatism and mean refractive error between the iris claw and the Carlevales’s IOL implant groups, probably due to the size of the corneal incision, which is roughly 6 mm for the iris claw and 2.2 mm for the Carlevales’s IOL, resulting in more significant postoperative induced astigmatism for the iris claw group [32].

## Conclusion

In conclusion, sutureless scleral fixation Carlevale IOL appears as a viable choice for the treatment of aphakia, IOL–bag complex dislocation and lens subluxation, due to its unique stability features. The lack of tactile manipulation, self-centration, and lens firm fixation are all benefits of this approach. This allows to reduce high order aberration such as astigmatism and coma, with a very good postoperative BCVA.

Additional research with more patients and longer follow-ups are surely required to confirm these preliminary findings.
